# Sex-specific changes in triglyceride profiles in liver cirrhosis and hepatitis C virus infection

**DOI:** 10.1186/s12944-022-01715-w

**Published:** 2022-10-24

**Authors:** Georg Peschel, Jonathan Grimm, Martina Müller, Marcus Höring, Sabrina Krautbauer, Kilian Weigand, Gerhard Liebisch, Christa Buechler

**Affiliations:** 1 Department of Internal Medicine I, Gastroenterology, Hepatology, Endocrinology, Rheumatology and Infectious Diseases, University Hospital Regensburg, 93053 Regensburg, Germany; 2 Department of Internal Medicine, Klinikum Fürstenfeldbruck, 82256 Fürstenfeldbruck, Germany; 3 Institute of Clinical Chemistry and Laboratory Medicine, Regensburg University Hospital, 93053 Regensburg, Germany; 4 Department of Gastroenterology, Gemeinschaftsklinikum Mittelrhein, 56073 Koblenz, Germany

**Keywords:** Genotype, Direct acting antivirals, Liver cirrhosis, Fibrosis-4 score, Polyunsaturated triglycerides

## Abstract

**Background:**

Hepatitis C virus (HCV) infection is associated with serum lipid abnormalities, which partly normalize following direct-acting antiviral (DAA) therapy. Here, associations of serum triglycerides (TGs) with viral genotype and markers of liver disease severity were evaluated in patients with chronic HCV.

**Methods:**

The study included the serum of 177 patients with chronic HCV. TGs were quantified by flow injection analysis Fourier transform mass spectrometry. Laboratory values and noninvasive scores for liver fibrosis assessment were determined. The nonparametric Kruskal‒Wallis test, one-way ANOVA, multiple linear regression and Student’s t test were used as appropriate. *P* values were adjusted for multiple comparisons.

**Results:**

HCV-infected women had lower serum TGs than men, and thus, a sex-specific analysis was performed. None of the 46 TG species analyzed differed in the serum of female patients with and without liver cirrhosis. In contrast, in the serum of male patients with liver cirrhosis, TGs with 53, 56 and 58 carbon atoms and three to eight double bonds were diminished. These polyunsaturated TGs were also low in males with a high fibrosis-4 score. TGs with 7 or 8 double bonds negatively correlated with the model of end-stage liver disease score in males. In addition, TGs with 49, 51 and 53 carbon atoms were reduced in male patients infected with genotype 3a in comparison to genotype 1a. TGs with 56 carbon atoms were lower in genotype 3a-infected males than in genotype 1b-infected males. TGs did not differ in females by genotype. Genotype 3-related changes disappeared at the end of therapy with DAAs. Overall, the levels of serum TGs did not change during DAA therapy in either sex. Consequently, the serum TGs of males with liver cirrhosis were lower than those of males without cirrhosis at the end of therapy. Such a difference was not apparent in females.

**Conclusions:**

The decline in TGs observed only in male patients with liver cirrhosis and male patients infected with genotype 3 illustrates sex-specific changes in lipid metabolism in chronic HCV.

**Supplementary Information:**

The online version contains supplementary material available at 10.1186/s12944-022-01715-w.

## Introduction

Chronic hepatitis C virus (HCV) infection has a worldwide prevalence of approximately 2.5%. HCV infection is a major cause of chronic liver diseases, liver cirrhosis and hepatocellular carcinoma [1]. An HCV vaccine is still not available [2]. Since 2014, patients can be treated with direct acting antivirals (DAAs) with cure rates of up to 100% within a treatment duration of 8 to 12 weeks [3, 4]. DAA therapy is similarly effective in patients without and with liver cirrhosis and in patients with different viral genotypes [3–5]. Elimination of HCV is associated with a decline in inflammatory cytokine and chemokine levels in blood [6–8]. The liver function of patients with cirrhosis and a model for end-stage liver disease (MELD) score ≥ 10 did not improve during a four-year follow-up after DAA treatment [5].

HCV uses the hosts´ lipid metabolism for infection, and it has been consistently reported that the serum low-density lipoprotein (LDL) of HCV patients is low [9, 10]. Accordingly, efficient elimination of HCV is related to recovery of systemic LDL levels shortly after the start of DAA treatment [3, 11–13]. In the blood, triglyceride (TG)-rich very low-density lipoprotein (VLDL) is converted to LDL by the activity of lipases, which hydrolyze TGs [14]. VLDL particles are TG-rich lipoproteins secreted by the liver that contain apolipoprotein B-100. HCV impairs the activity of microsomal triglyceride transfer protein and thus loading of apolipoprotein B-100 with TGs [15].

Unexpectedly, DAA therapy did not significantly influence serum TG levels [3]. Similarly, serum TG levels were comparable between HCV-infected patients and noninfected controls [16, 17]. The TG content of VLDL particles can be directly measured and was indeed reduced in HCV infection. Non-VLDL-TG levels were higher in the serum of HCV patients, and thus total TGs did not change [17]. A decline in VLDL-TGs in HCV infection was reported by a separate study, and TGs in high-density lipoprotein (HDL) increased [18].

Reports on the effect of DAA treatment on blood TGs are, however, contradictory. An increase in blood TGs was described in some studies [19–21], whereas other studies could not identify a change in TG levels by DAA therapy [3, 22, 23] or even observed a decline [24, 25]. It was also reported that elimination of HCV led to a rise in serum TGs in HCV patients with fibrosis stages F0-F2 but not in those with advanced disease [21, 26]. Thus, differences between the study groups regarding fibrosis stage may account for the discrepant findings. The viral genotype may also have a role herein, and a less prominent rise in TGs after viral clearance was found in genotype 2 compared to genotype 1 infected patients [27].

A cross-sectional analysis of nearly 12,000 adults showed that individuals with high fibrosis-4 (FIB-4) scores had lower fasting TGs [28]. Notably, total serum TGs and VLDL-TGs were reduced in patients with histological liver cirrhosis compared to healthy controls and to patients with fibrosis stages 1 to 3 [17]. Advanced liver fibrosis was also found to be associated with high TGs in LDL and HDL and low VLDL-TGs, whereas total serum TGs did not change [18]. In separate cohorts of HCV patients, TG levels were not affected by cirrhosis [20, 21, 26] or positively correlated with the FIB-4 score [29].

Overall, men have higher serum TGs than females [30]. Most of the studies published thus far did not perform sex-specific analysis. Notably, in a cohort of patients without viral hepatitis, the inverse association between TG levels and fibrosis severity was stronger in men [28]. Thus, associations of serum TGs with liver cirrhosis may be affected by sex.

Moreover, in the studies listed above, total TG levels were measured, although many different TG species circulate in blood [31]. Certain lipid species within the same lipid class were found to be associated with poorer metabolic health and others with better metabolic health [32]. In experimental nonalcoholic fatty liver disease (NAFLD), plasma TG species with fewer double bonds were linked to NAFLD [32]. The diabetes risk of humans was associated with TG species having fewer double bonds and shorter acyl chains [33]. Thus, the total concentration of TGs and the TG composition are associated with disease states.

Here, it was hypothesized that serum TG concentrations and eventually the TG species profile differ between males and females with chronic HCV infection and that TGs are low in patients with liver cirrhosis. DAA therapy does not improve liver function in the short term, suggesting that TG levels and TG composition are similar before therapy and at the end of therapy. The objective of the investigation was to measure 46 TG species differing by carbon chain length and/or double bond number in the serum of 177 patients with chronic HCV and to perform a sex-specific analysis for correlations with markers of disease severity. It was also the aim to show that DAA therapy does not change TG levels or TG composition.

## Materials and methods

### Study cohort

This study was conducted from October 2014 to September 2019 at the Department of Internal Medicine I (University Hospital of Regensburg) [7]. HCV treatment naïve patients with chronic disease were enrolled in the study. DAA therapy is overall well tolerated. Usually, two DAAs belonging to different drug classes are combined, and almost any patient can now be successfully treated [34, 35]. In line with this, all of the patients were appropriate for DAA therapy according to recent guidelines [36]. The patients were older than 18 years and were not coinfected with hepatitis B virus or human immunodeficiency virus. Patients signed an informed consent form. DAAs (sofosbuvir/daclatasvir, sofosbuvir/ledipasvir, sofosbuvir/velpatasvir, glecaprevir/pibrentasvir, or elbasvir/grazoprevir) in accordance with international treatment guidelines [36] were given to the patients for 12 weeks. None of the patients died during the study or discontinued participation in the study. There were very few patients taking statins. Drug‒drug interactions between DAAs and statins were reported [37], and thus, statin therapy was suspended during DAA therapy.

Laboratory parameters were measured by the Institute of Clinical Chemistry and Laboratory Medicine (University Hospital Regensburg).

Cirrhosis diagnosis by ultrasound determined the liver surface and the size of the liver and liver parenchyma [38]. The cutoff values used for the fibrosis-4 (FIB-4) scoring were as follows: no fibrosis: < 1.30 for patients younger than 65 years and < 2.00 for patients older than 65 years, and a score of > 3.25 was defined as advanced fibrosis [39]. Laboratory values were available for at least 95% of the patients.

### Quantification of triglyceride species

Serum triglyceride species were quantified by flow injection analysis Fourier transform mass spectrometry (FIA-FTMS) as described previously [40]. Total TG levels were the sum of all TG species measured in the serum of the patients.

### Statistical analyses

Data are shown as boxplots, which give the minimum, the maximum, the median, and the first and third quartiles. Small circles or asterisks above or below the boxes mark outliers. When more than one TG class is shown in a figure, mean concentrations ± standard deviations are displayed. Data in tables are given as median values, and the minimum and maximum values in brackets.

The nonparametric Kruskal‒Wallis test and one-way ANOVA were used for comparison of continuous variables between independent groups. Stepwise multiple linear regression analysis was also performed. Spearman correlation was used for correlation analysis (SPSS Statistics 25.0 program). Student´s t test was used to analyze paired data (Microsoft Excel). *P* values were adjusted for multiple comparisons by Bonferroni. A value of *P* < 0.05 was regarded as significant.

## Results

### Association of TG species levels with sex, fatty liver, diabetes, age and body mass index in HCV patients

**Fig. 1:**
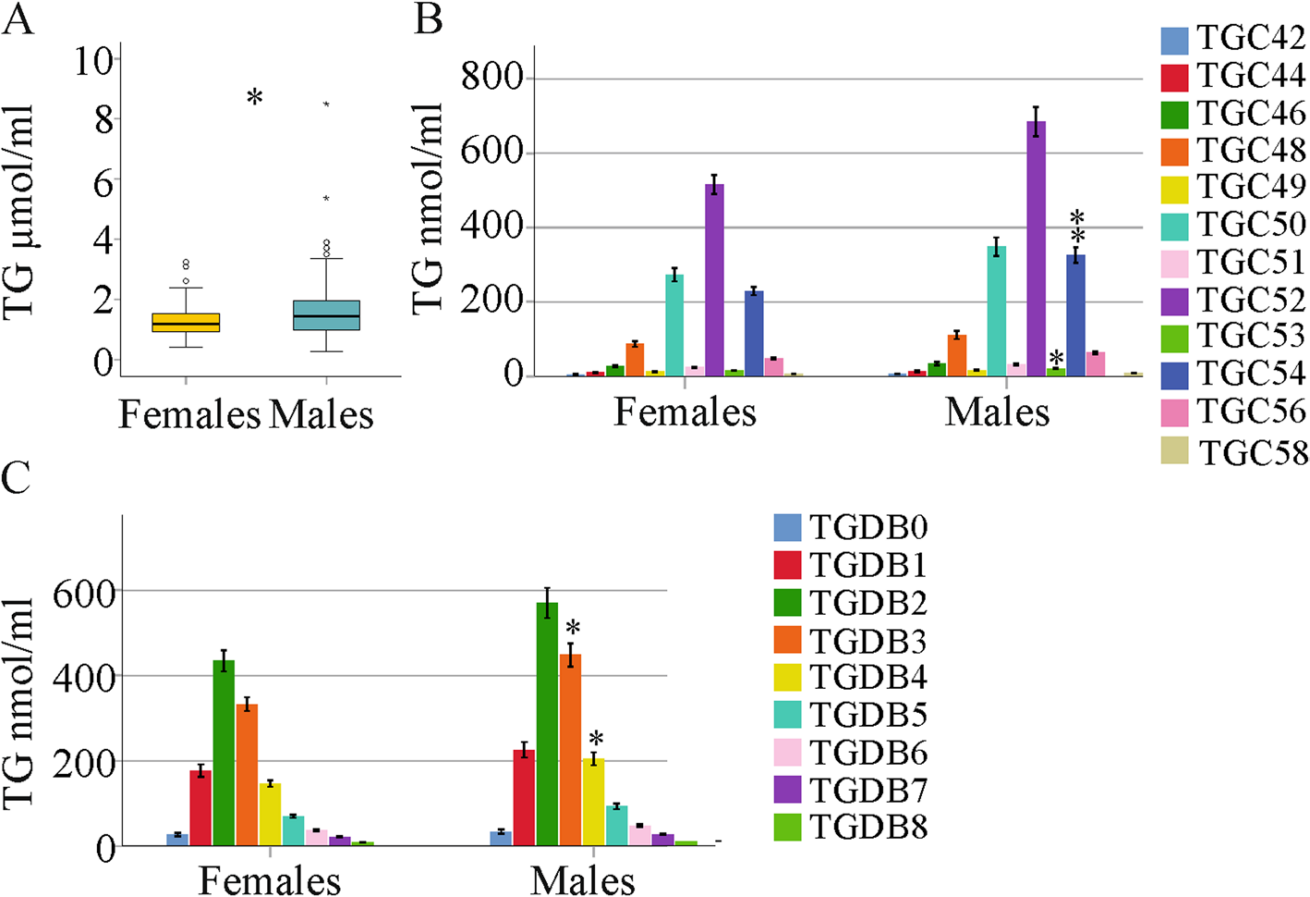
Serum TG species in relation to sex in patients with chronic HCV. (**a**) Total TGs in female and male patients (median level and range); (**b**) TG species with an identical number of carbon atoms were grouped together and compared between female and male patients (mean concentration ± standard deviation); (**c**) TG species with an identical number of double bonds were grouped together and compared between female and male patients (mean concentration ± standard deviation). * *P* < 0.05, ** *P* < 0.01

Triglycerides are neutral lipids, and their blood content and composition are regulated by different pathways [41]. Here, 46 TG species differing in carbon length and/or double bond number were measured in the serum of 177 patients with chronic HCV. For analysis, TGs with an identical number of carbon atoms (C42 – C58) or number of double bonds (DB0 – 8) were grouped together. Total serum TGs were higher in males than females (Fig. 1a). TGs with 53 or 54 carbon atoms and with 3 or 4 double bonds were significantly elevated in male serum (Fig. 1b, c). Given that most TG species tended to be higher in males than females, the distribution of TG species relative to total TG levels did not vary between the groups (*P* > 0.05 for all).

The 103 male patients had a higher model of end-stage liver disease (MELD) score, alanine aminotransferase (ALT), aspartate aminotransferase (AST) and international normalized ratio (INR) and lower high-density lipoprotein (HDL) levels than the 74 female patients (Table 1). Platelets, bilirubin, albumin, creatinine, leukocytes, C-reactive protein (CRP), LDL, age and body mass index (BMI) were similar in both sexes (Table 1).

**Table 1:** Laboratory parameters of all patients and of patients with liver cirrhosis at baseline separated by sex

Laboratory parameter	Females (74 patients)	Males (103 patients)	*P*-value	Females – Cirrhosis (16 patients)	Males – Cirrhosis (24 patients)	*P*-value
Age years	56 (24–79)	52 (25–82)	ns	64 (52–79)	57 (38–80)	ns
BMI kg/m^2^	25.4 (17.6–41.6)	25.7 (17.9–37.4)	ns	25.4 (21.2–41.6)	26.8 (19.6–37.4)	ns
MELD	7 (6–20)	7 (6–21)	0.032	9 (7–20)	9 (7–19)	ns
Platelets n/nl	209 (47–364)	185 (38–402)	ns	85 (47–282)	92 (38–282)	ns
ALT U/l	56 (19–232)	82 (24–305)	< 0.001	59 (27–139)	78 (31–240)	ns
AST U/l	43 (7–208)	57 (15–1230)	0.006	69 (7–208)	78 (36–148)	ns
Bilirubin mg/dl	1.0 (1.0–2.4)	1.0 (1.0–4.3)	ns	1.0 (1.0–2.4)	1.1 (1.0–4.3)	ns
Albumin g/l	39 (24–46)	38 (19–45)	ns	35 (24–43)	34 (19–45)	ns
INR	1.04 (1.00–2.44)	1.06 (1.00–2.33)	0.049	1.18 (1.00–2.44)	1.26 (1.02–2.33)	ns
Creatinine mg/dl	1.00 (1.00–1.73)	1.00 (1.00–4.00)	ns	1.00 (1.00–1.73)	1.00 (1.00–1.31)	ns
Leukocytes n/l	6.4 (3.0–12.6)	6.5 (2.2–72.4)	ns	5.1 (3.0–12.6)	5.5 (2.6–9.9)	ns
CRP mg/l	2.9 (2.9–55.0)	2.9 (2.8–14.4)	ns	2.9 (2.9–4.4)	2.9 (2.9–9.1)	ns
HDL mg/dl	57 (27–111)	49 (19–103)	< 0.001	47 (27–78)	52 (26–103)	ns
LDL mg/dl	88 (33–179)	100 (23–219)	ns	58 (33–133)	82 (31–152)	ns

*ALT* Alanine aminotransferase, *AST* Aspartate aminotransferase, *BMI* Body mass index, *CRP* C-reactive protein, *HDL* high-density lipoprotein, *LDL* Low-density lipoprotein, *ns* not significant

**Fig. 2:**
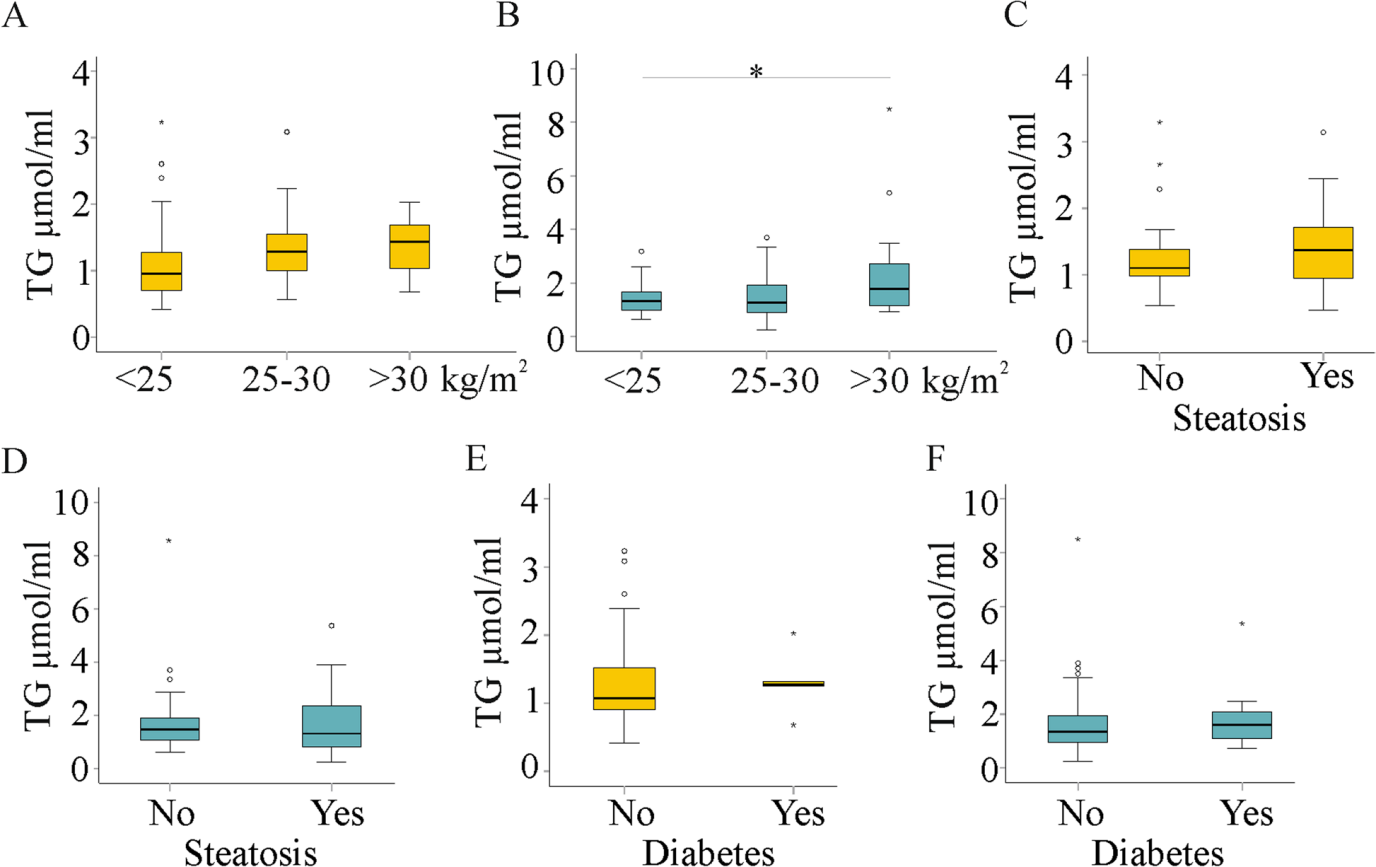
Serum TG species in relation to body mass index (BMI) in female and male patients with chronic HCV. (**a**) Total TGs in female patients stratified for BMI; (**b**) Total TGs in male patients stratified for BMI; (**c**) Total TGs in female patients with and without liver steatosis; (**d**) Total TGs in male patients with and without liver steatosis; (**e**) Total TGs in female patients with and without diabetes; (**f**) Total TGs in male patients with and without diabetes. * *P* < 0.05

TGs increased with higher BMI in males but not in females (Fig. 2a, b). Several TG species were elevated in obese men, and a significant increase was noted for TG C54, TG DB1 and TG DB4 (Figure S1). Relative levels of the TGs did not differ between the normal-weight and obese men, showing that all TGs increased in parallel (*P* > 0.05). Seventy-three patients had fatty liver (38 males and 35 females), and 20 patients had diabetes (15 males and 5 females), but this was not associated with higher concentrations of TGs (Fig. 2c-f). None of the TGs correlated with BMI or age in females or males (Table S1 and S2).

### Association of TG species levels with parameters of liver function and lipoprotein levels in HCV patients before DAA therapy

In the female patients with HCV, none of the TG species correlated with the MELD score. There were no associations with albumin, bilirubin, AST, ALT, platelets, creatinine, CRP or leukocytes (Table S1). In males, a negative correlation of the MELD score with TG C56, C58, DB7 and DB8 was identified (Table S2). The INR negatively correlated with TG C53, C56, C58, DB7 and DB8 (Table S2). There were no associations with albumin, bilirubin, AST, ALT, platelets, creatinine, CRP or leukocytes (Table S2).

As expected, serum TGs correlated with LDL and HDL. In females, negative correlations of HDL with TG C46, C48, C49, C50, C51, C52, C53, DB0, DB1, DB2, DB3 and total TG levels were found (Table S1). In males, associations between HDL and TG C48, C50, C51, C52, C53, C54, DB0—DB6 and total TG levels existed (Table S2). Positive associations of TGs with LDL were found for all but TG C42, C44, C46, C54, DB0 and DB1 in males. In females, TG C52, C56, C58, DB2 to DB8 and total TG levels positively correlated with LDL (Table S1 and S2).

### Association of TGs with noninvasive scores of liver fibrosis and ultrasound-diagnosed cirrhosis in HCV patients

**Fig. 3:**
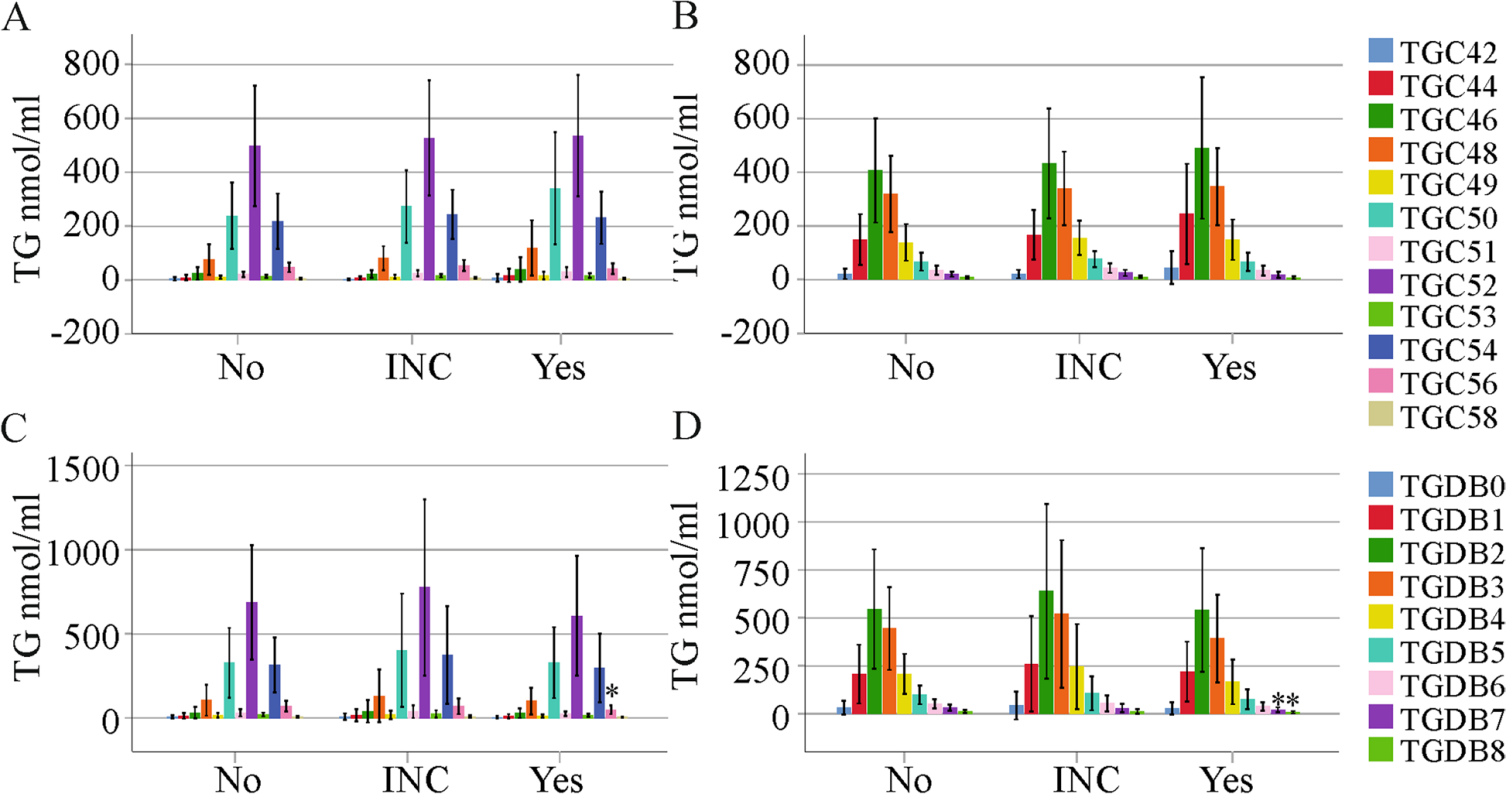
TGs in relation to the FIB-4 score. (**a**) TGs with an identical number of carbon atoms in females with no (33 patients), inconclusive (INC, 24 patients) and definite fibrosis (Yes, 17 patients); (**b**) TGs with identical double bonds in females with no, inconclusive and definite fibrosis; (**c**) TGs with an identical number of carbon atoms in males with no (42 patients), inconclusive (28 patients) and definite fibrosis (33 patients); (**d**) TGs with identical double bonds in males with no, inconclusive and definite fibrosis. * *P* < 0.05 for comparison of No and Yes

The FIB-4 score is a noninvasive measure of liver disease severity in HCV [42]. In female HCV patients, none of the TGs changed with increasing FIB-4 scores (Fig. 3a, b). In males, TG C56, DB7 and DB8 were reduced in patients with fibrosis compared to those with no liver fibrosis (Fig. 3c, d and Table S3).

**Fig. 4:**
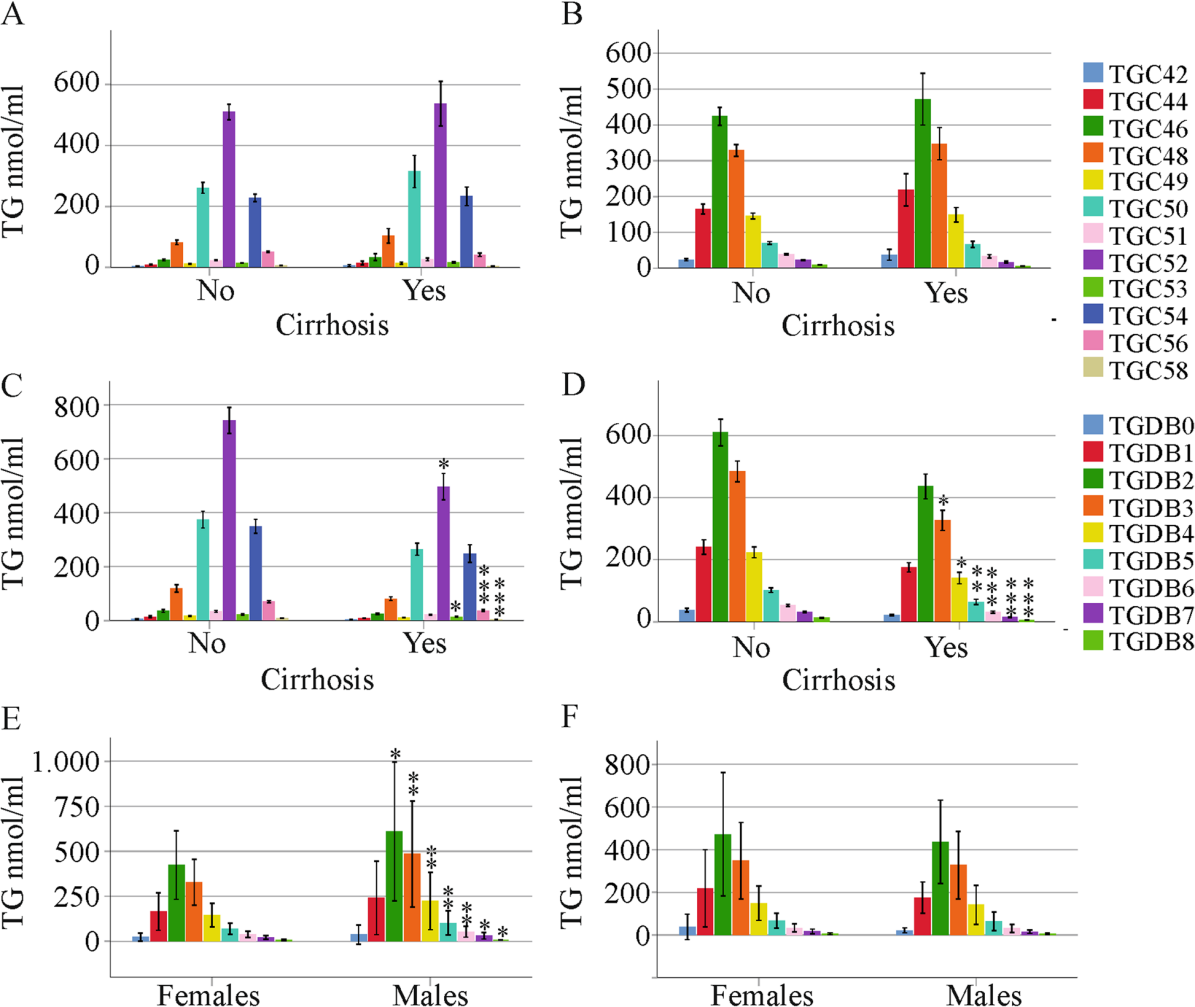
TGs in relation to cirrhosis diagnosed by ultrasound. (**a**) TGs with an identical number of carbon atoms and (**b**) TGs with identical double bonds in females without (No) and with (Yes) liver cirrhosis diagnosed by ultrasound; (**c**) TGs with an identical number of carbon atoms and (**d**) TGs with identical double bonds in males without (No) and with (Yes) liver cirrhosis diagnosed by ultrasound; (**e**) TGs with an identical number of carbon atoms in males and females without liver cirrhosis; (**f**) TGs with an identical number of carbon atoms in males and females with liver cirrhosis. * *P* < 0.05, ** *P* < 0.01, *** *P* < 0.001

Along the same lines, none of the TGs declined in the 16 females where cirrhosis was diagnosed by ultrasound (Fig. 4a, b). In males, TG C52, C53, C56, C58, DB3, DB4, DB5, DB6, DB7 and DB8 were low in the 24 patients with liver cirrhosis (Fig. 4c, d). Significant declines in percent (when all TGs were set to 100%) TG C56 (*P* < 0.001), TG C58 (*P* = 0.01), TG DB6 (*P* = 0.02), TG DB7 (*P* < 0.001) and TG DB8 (*P* < 0.001) showed that these species were specifically low in males with liver cirrhosis. Total TGs were reduced in male patients with liver cirrhosis compared to males without cirrhosis (*P* = 0.019).

Whereas TGs (TG DB2 – DB8) were higher in males than in females in the cohort of patients without liver cirrhosis, this difference was no longer apparent in patients with liver cirrhosis (Fig. 4e, f). Notably, serum lipoprotein levels and measures of liver injury did not differ by sex in the cirrhosis group (Table 1).

### Association of TGs with viral load and genotype

**Fig. 5:**
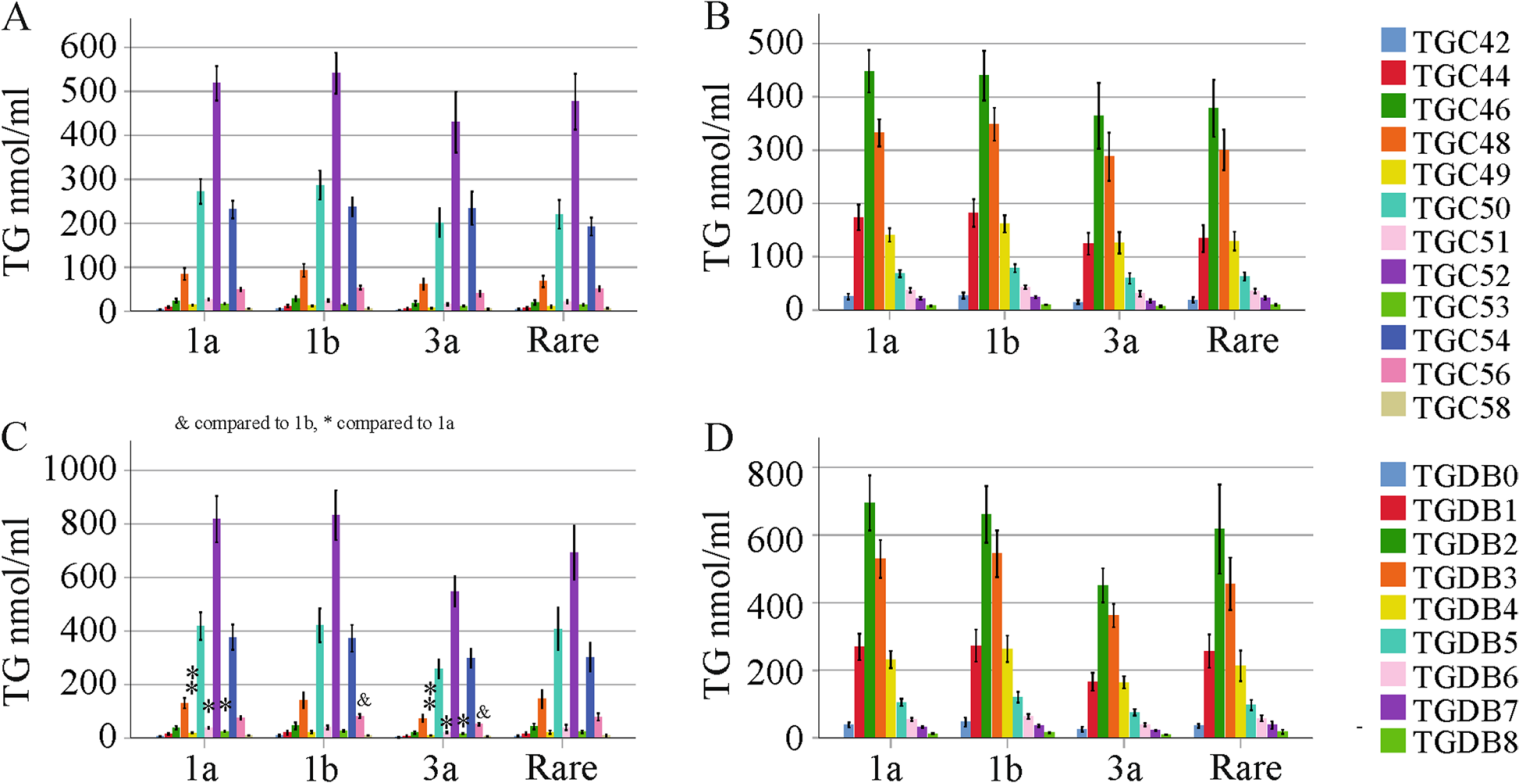
TGs stratified for viral genotypes in patients without liver cirrhosis. (**a**) TGs with an identical number of carbon atoms and (**b**) TGs with identical double bonds in females with different viral genotypes; (**c**) TGs with an identical number of carbon atoms and (**d**) TGs with identical double bonds in males with different viral genotypes. * *P* < 0.05, ** *P* < 0.01, & *P* < 0.05

None of the TG species in male or female serum correlated with viral load (Table S1 and S2). In females, viral genotypes were 1a in 22, 1b in 32, 3a in 10 and rare genotypes such as 2a and 2b in 10 patients. In males, genotypes were as follows: 30 patients with 1a, 44 patients with 1b, 25 patients with 3a and 4 patients with rare genotypes. Because of the decline in TGs in male patients with cirrhosis (Fig. 4c, d), associations with genotypes were analyzed in the noncirrhosis patients. Viral genotype had no effect on TG distribution in female serum (Fig. 5a, b). In males, TG C49, C51 and C53 were lower in genotype 3a-infected patients than in 1a-infected patients, and TG C56 was reduced in 3a-infected males compared to 1b-infected males (Fig. 5c and Table S4). TGs stratified by the number of double bonds did not differ by genotype in males (Fig. 5d). Total serum TG levels were similar between the genotypes in males and females (*P* = 0.064 for males and *P* = 0.576 for females). Moreover, serum HDL (*P* = 0.069) and LDL (*P* = 0.063) did not significantly decline in genotype 3a-infected males.

### TGs at 4 and 12 weeks after the start of therapy with direct acting antivirals

**Fig. 6:**
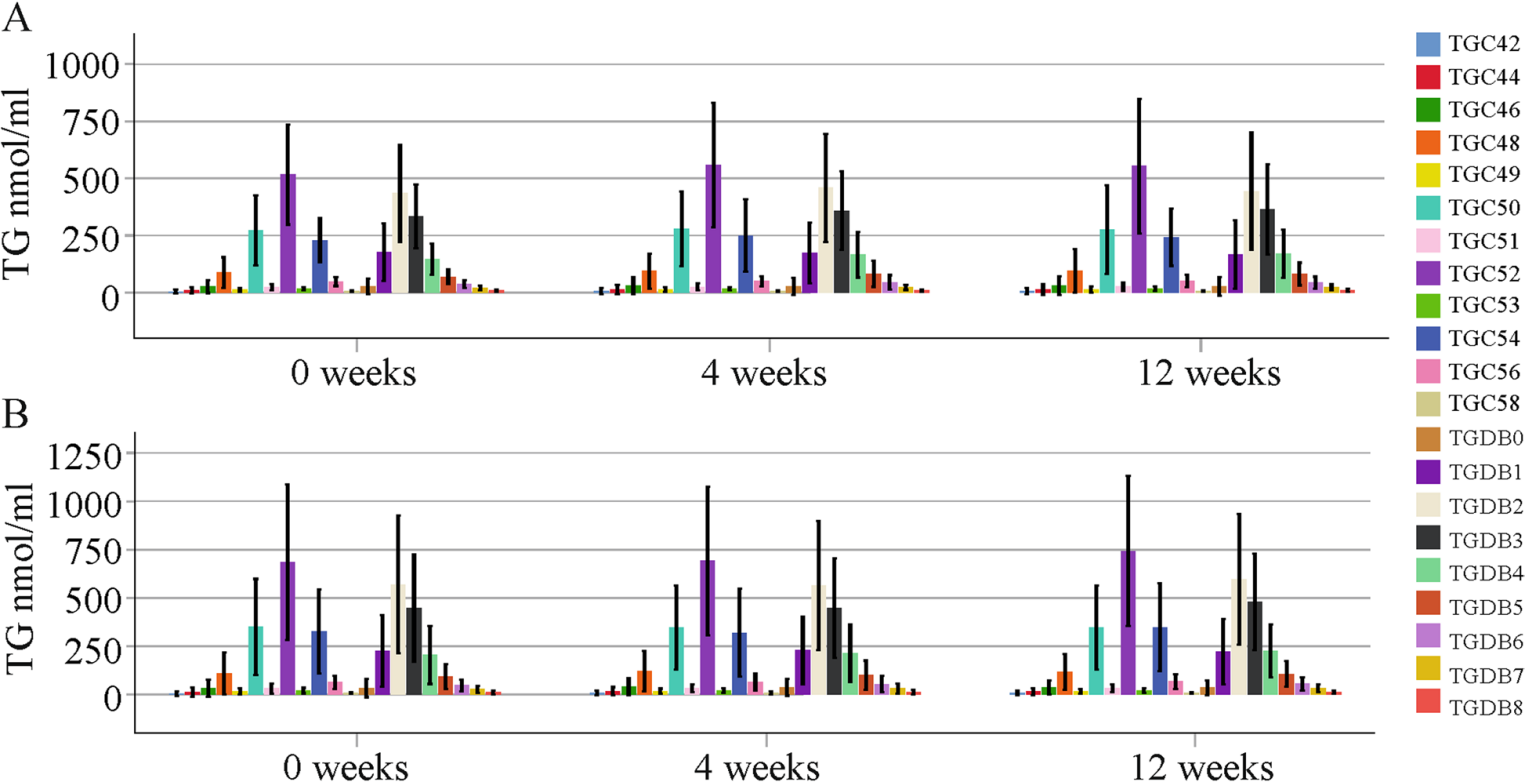
TG levels during the study. (**a**) TG levels during the study in females; (**b**) TG levels during the study in males

At 4 and 12 weeks after the start of therapy, TG levels were not changed in females or males compared to pretreatment concentrations (Fig. 6). This was also the case when male and female patients with and without cirrhosis were separately analyzed (data not shown).

**Fig. 7:**
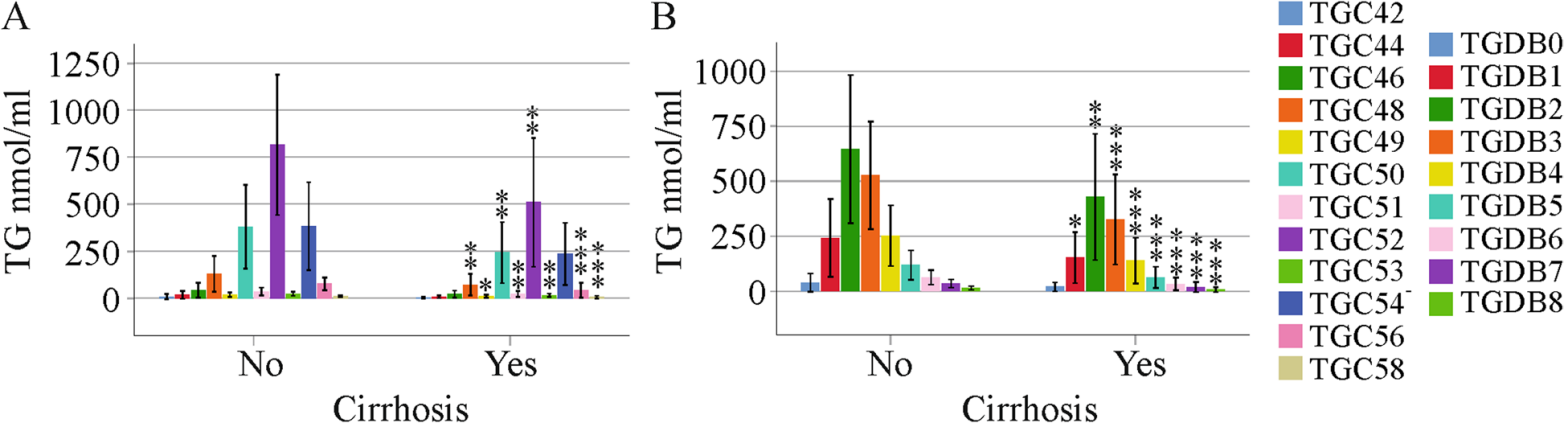
TGs in male patients at the end of therapy. (**a**) TGs with an identical number of carbon atoms in males without (No) and with (Yes) ultrasound diagnosed liver cirrhosis; (**b**) TGs with identical double bonds in males without and with ultrasound diagnosed liver cirrhosis. * *P* < 0.05, ** *P* < 0.01, ^***^ *P* < 0.001

At the end of therapy, TGs did not differ between female patients with and without liver cirrhosis (data not shown). TG C48 to C58 and TG DB1 to DB8 were reduced in male patients with ultrasound-diagnosed liver cirrhosis compared to males without cirrhosis (Fig. 7). The percent TG C56 (*p* = 0.015), TG DB5 (*p* = 0.015), TG DB6 (*p* = 0.01w), TG DB7 (*p* = 0.002) and TG DB8 (*p* = 0.006) of total TG levels were low in males with cirrhosis compared to males without cirrhosis.

In females, associations of TGs with measures of liver injury did not exist (Table S5). In males, TG C56 and TG DB4 to DB8 negatively correlated with the MELD score. TG C56, TG DB3 to DB8 and total TGs correlated negatively with INR. TG C56, C58 and TG DB5 to DB8 were positively related to serum albumin. ALT correlated with TG C56, C58, TG DB7 and DB8 (Table S6). Importantly, none of the associations of TGs with the MELD score were significant after adjusting for LDL levels (Table S7).

To analyze whether the MELD score, albumin and ALT (which were correlated with serum levels of TG C56, TG DB7 and DB8 in males (Table S6)) contribute to the serum levels of these TG species, a stepwise multiple linear regression analysis was conducted. This analysis showed that albumin, but not the MELD score or ALT, was independently associated with the serum levels of these TG species. Albumin was significantly related in males to TG C56 as the dependent variable (*R*^2^ = 0.081, the regression coefficient B = 2.491 and *P* = 0.002), to TG DB7 as the dependent variable (*R*^2^ = 0.076, the regression coefficient B = 1.331 and *P* = 0.003), and to TG DB8 as the dependent variable (*R*^2^ = 0.062, the regression coefficient B = 0.551 and *P* = 0.007).

At the end of DAA therapy, none of the TGs correlated with age in males. In females TG C42, 44, 46, 48, 49, 50, 51, DB0 and DB1 were positively associated with age (Table S5 and S6).

TG C50 to C54 and DB1 to DB5 as well as total TG levels negatively correlated with HDL, and all but TG42 correlated positively with LDL in males. In females, none of the TGs was associated with LDL, and TG C50, C52, C54, and TG DB2 to DB7 and total TGs were negatively correlated with HDL (Table S5 and S6).

Notably, TG levels in males without liver cirrhosis were similar between the genotypes at the end of the study (Figure S2).

## Discussion

Our study shows that serum TGs are reduced in male HCV patients with liver cirrhosis in comparison to male patients without cirrhosis. Such a difference was absent in females with chronic HCV. Notably, men had higher TGs than females in the cohort of patients without liver cirrhosis, and this difference disappeared in the cirrhosis group. Thus, HCV cirrhosis-related abnormalities in TG metabolism mostly affect male patients.

HCV infection is well described to lower serum LDL in both sexes, and LDL recovers at approximately 4 weeks after the start of DAA treatment [11, 12, 20]. Approximately 50% of the lipids of LDL are free cholesterol and cholesteryl esters [43], and cholesterol is induced shortly after virus elimination [44]. Serum TG levels do not change during DAA therapy, which is in line with several published studies [3, 22, 23]. TGs in serum are most abundant in chylomicrons and VLDL and make up less than 10% of the lipids carried in LDL or HDL [43]. Whether VLDL-TG levels and non-VLDL-TG levels change during DAA treatment has not been studied thus far.

HCV particles associate with triglyceride-rich lipoproteins during their morphogenesis. Production of these lipo-viral particles uses the pathways involved in VLDL production and interferes with VLDL assembly and secretion [45], and this is most evident in genotype 3 HCV [45]. In the cohort studied, serum TGs did not correlate with viral load and were not changed at the end of therapy. Regarding viral genotype, a sex-related effect was observed in males. Although the total levels of TGs were similar between genotypes, less abundant TG species declined in male patients infected with genotype 3a compared to genotype 1. Importantly, these genotype 3a-related changes in TGs disappeared at the end of DAA treatment, suggesting that this is a specific effect of HCV genotype 3a infection in males.

Men have higher serum TG levels than females [30], and this also applies to HCV-infected patients. Here, TG C53, C54, DB3 and DB4 were significantly increased in the serum of male patients. The median serum TG levels in males were approximately 1400 nmol/ml, TG C54 had a median concentration of 280 nmol/ml and TG DB3 made up approximately 400 nmol/ml, suggesting that relatively common TG variants are higher in males. However, because of a modest elevation of all TG species in males, the distribution of TG species was similar between males and females.

Sex differences in lipid metabolism, pathology of chronic liver diseases, fibrosis progression and HCC have been well described. These differences can be attributed to gonadal steroid hormones [46], higher iron levels in the liver of male patients [47] and better immune response of females [48].

Females have more adipose tissue mass, which releases more glycerol into the circulation. Despite having increased plasma glycerol, hepatic glycerol uptake and subsequently systemic triglycerides, are lower in women than men [49]. Plasma glycerol levels are indeed increased in cirrhosis patients [50]. Hepatic glycerol uptake and/or lipolysis in fat tissues may be affected in males rather than females with liver cirrhosis, which may cause a decline in systemic TG levels in males. Insulin-mediated suppression of lipolysis is less effective in men and is a further reason for higher circulating TGs [51]. Insulin resistance is relatively common in patients with liver cirrhosis [52] and may contribute to higher free fatty acid and glycerol levels in the circulation [50]. Sex-related differences in these pathways are mostly not well defined. TGs decline in HCV-infected men with liver cirrhosis, but the exact mechanisms that play a role are not yet understood.

Multiple linear regression analysis revealed that 8.1%, 7.6% and 6.2% of serum TG C56, DB7 and DB8, respectively, in males were related to albumin, whereas the MELD score and ALT were irrelevant. Albumin homeostasis is regulated by hepatic synthesis and its removal by renal, gastrointestinal and catabolic processes [53]. The mechanisms connecting albumin and polyunsaturated TG levels remain to be clarified.

Overweight/obesity is associated with increased blood TG levels in both sexes [54–56]. In HCV patients, a significant rise in TGs in obese individuals was found in males, and TG C54, DB1 and DB4 were induced. Nearly all TG isoforms were elevated in the serum of obese males (up to 320% higher in the obese than the normal weight) and females (up to 508% higher in the obese than the normal weight), but this did not reach significance. This suggests that obesity is associated with higher serum triglycerides in HCV in both sexes, and this finding meets almost all of the TG species analyzed.

Obesity is a risk factor for fatty liver and diabetes [57, 58], but none of the TGs were changed in the serum of the patients with these comorbidities. In accordance with the current observation, Valkov et al. reported that serum triglycerides were not associated with steatosis in HCV [29]. The number of patients with diabetes may have been too small to identify differences.

Blood TGs of adults increase with age [59]. Associations of age with TGs were only identified in females at the end of therapy. Notably, TGs with fewer carbon atoms and having no or just one double bond positively correlated with age. Higher age is associated with an increased risk for metabolic diseases [59, 60], and lipids with shorter acyl chain length and higher degree of saturation may have a role herein [33].

Associations of serum TGs and lipoprotein levels in healthy individuals have been shown before [61]. It should be noted that serum TGs correlated negatively with HDL and positively with LDL before the start of therapy and at the end of therapy in males. In females, such associations were observed before treatment. At the end of therapy, a positive correlation of LDL and TGs did not exist. HDL and LDL composition are highly diverse, and TG content of the different particles can vary up to twofold [62, 63]. Although TG levels in serum do not change during DAA treatment, it is well possible that lipoprotein composition and thus the biologic function [62, 64] of these particles is affected.

In women, serum TGs did not change in cirrhosis, which was diagnosed by ultrasound. Correlations with markers of liver injury did not exist. Notably, TGs declined in men with ultrasound-defined liver cirrhosis, and the sex-based differences in serum TG levels disappeared. Negative associations of serum TG levels with liver disease severity have been described before. A cross-sectional analysis showed that a high FIB-4 score was related to low fasting triglycerides [28]. Serum TGs and serum VLDL-TGs were found to be reduced in patients with liver cirrhosis in comparison to healthy controls and to patients with less severe stages of liver fibrosis [17]. Although there is accumulating evidence that lipid metabolism is different between males and females, most of the studies published did not perform sex-specific analysis. Our investigation showed that serum TGs are low in men with liver cirrhosis in comparison to men without cirrhosis, and this is the case before therapy start and at therapy end.

Notably, in men with ultrasound-diagnosed liver cirrhosis, TGs with 53, 56 and 58 carbon atoms and three or more double bonds declined. In males, TG C56 and C58 as well as TGs with seven or eight double bonds negatively correlated with the MELD score before therapy start. At the end of therapy, TG C56 and TGs with four or more double bonds were negatively associated with the MELD score. In cirrhosis defined by a high FIB-4 score, TG C56, TG DB7 and DB8 were low in male patients with liver cirrhosis in comparison to patients without advanced fibrosis.

Noninvasive scores of liver cirrhosis as well as liver biopsies have limitations [65], and a combination of serum and imaging techniques improves their accuracy [66]. Thus, it is likely that at least TG C56, TG DB7 and TG DB8 are low in male HCV patients with liver cirrhosis.

The use of different techniques and scores to diagnose liver cirrhosis may also explain why not all studies described a decline in TGs in cirrhosis [29]. HCV patients (four of the five patients were males) with decompensated liver cirrhosis had lower levels of polyunsaturated fatty acids in VLDL-TG than healthy controls (three males, six females) [67]. Although this study may be biased because of sex differences between patients and controls, the lower levels of polyunsaturated fatty acids in the serum of male patients with liver cirrhosis [67] are principally in accordance with the lower levels of polyunsaturated TGs observed in the current cohort.

The more saturated TG species do not significantly change in cirrhosis and do not correlate with the MELD score. The composition of the serum TG pool is different in males with liver cirrhosis. Reduced levels of lipids with longer acyl chains and a higher number of double bonds were associated with less metabolic health [33]. This may also apply to males with liver cirrhosis of HCV etiology. Dietary intake of omega-3 polyunsaturated fatty acids has positive effects for HCV patients [67, 68], and future studies may more precisely delineate these benefits for both sexes.

## Comparisons with other studies and what does the current work add to the existing knowledge

Most of the studies analyzing TG levels in HCV patients measured total concentrations and did not discriminate TG species [3]. To the best of our knowledge, sex-related analysis of serum TG species in HCV has not been performed before. The current work provides evidence that viral genotype and liver cirrhosis are related to an altered TG profile in males but not in females. Genotype-related effects resolved at therapy end, whereas cirrhosis-associated changes persisted.

## Study strengths and limitations

The strength of this study is that a comparatively high number of different TG species were analyzed in the serum of HCV patients. The cohort was large enough to identify viral genotype- and sex-related differences. A limitation of this study is that healthy controls and patients with chronic liver diseases of different etiologies were not included. There were only a few diabetic patients, and the finding that TG species were not changed in diabetes cannot be extended to HCV-infected patients in general. TGs were measured before DAA treatment and at the end of therapy, and data for later time points were missing. Moreover, lipid-lowering drug intake was documented only for statins, and these were the only drugs suspended during DAA treatment. VLDL levels were not determined, and TGs were measured in serum and not in the individual lipoproteins. Patient blood was not obtained in the fasting state, and nonfasting serum was used for TG analysis. It is well known that serum TGs are higher in the postprandial state. While TG levels are nearly twofold increased after breakfast when the person has fasted overnight, the changes during the day are less pronounced [67, 69]. The current study suggests that nonfasting serum can be used to identify associations of serum TG with liver disease severity and sex. It cannot be excluded that the TG composition of fasting and nonfasting serum differs. However, the kind of nutrients affect TG levels and composition [70, 71] and most likely vary between patients, but this did not impede the identification of sex and disease-related changes.

## Conclusion

In summary, this study showed that the decline in polyunsaturated TG species in liver cirrhosis is male specific, as are HCV genotype 3-associated changes. These sex differences in TG levels may indicate that the disease pathophysiology and therapy of HCV-related liver cirrhosis differ in females and males. Thus, males may profit more from diets rich in polyunsaturated fatty acids than females.

## Supplementary Information

**Additional file 1:** **Table S1.** Spearman correlation coefficients for the correlations of TGs (where species with identical numbers of carbon atoms or double bonds were grouped together) with age, BMI, MELD score, viral load and routine laboratory parameters in female HCV patients before DAA therapy. **Table S2.** Spearman correlation coefficients for the correlations of TGs (where species with identical numbers of carbon atoms or double bonds were grouped together) with age, BMI, the MELD score, viral load and routine laboratory parameters in male HCV patients before DAA therapy. **Table S3.** TGs (where species with identical numbers of carbon atoms or double bonds were grouped together) in the serum of males before DAA therapy stratified by fibrosis-4 score. **Table S4.** TGs (where species with identical numbers of carbon atoms were grouped together) in the serum of males without liver cirrhosis before DAA therapy stratified by genotype. **Table S5.** Spearman correlation coefficients for the correlations of TGs (where species with identical numbers of carbon atoms or double bonds were grouped together) with age, BMI, the MELD score, viral load and routine laboratory parameters in female HCV patients at the end of DAA therapy. **Table S6.** Spearman correlation coefficients for the correlations of TGs (where species with identical numbers of carbon atoms or double bonds were grouped together) with age, BMI, MELD score, viral load and routine laboratory parameters in male HCV patients at the end of DAA therapy. **Table S7.** Spearman correlation coefficients for the correlations of TGs (where species with identical numbers of carbon atoms or double bonds were grouped together) with the MELD score at the end of DAA therapy in males. **Figure S1.** Serum TG species in relation to body mass index (BMI) in female and male patients with chronic HCV. **Figure S2.** TGs stratified according to viral genotypes in male patients without liver cirrhosis at the end of the study.

## Data Availability

Original data can be obtained from the corresponding author.

## Abbreviations

ALT: Alanine aminotransferase
AST: Aspartate aminotransferase
BMI: Body mass index
CRP: C-reactive protein
DAAs: Direct acting antivirals
FIB-4: Fibrosis-4
HCV: Hepatitis C virus
HDL: High-density lipoprotein
INR: International normalized ratio
LDL: Low-density lipoprotein
MELD: Model of end-stage liver disease
NAFLD: Nonalcoholic fatty liver disease
TGs: Triglycerides
VLDL: Very low-density lipoprotein
