# Supplementary material for: Sex-specific changes in triglyceride profiles in liver cirrhosis and hepatitis C virus infection
Source: Lipids Health Dis. 2022 Oct 24;21:106. doi: 10.1186/s12944-022-01715-w (PMC9590217; doi:10.1186/s12944-022-01715-w)
Supplement: Supplementary file 1 — Additional file 1: Table S1. Spearman correlation coefficients for the correlations of TGs (where species with identical numbers of carbon atoms or double bonds were grouped together) with age, BMI, MELD score, viral load and routine laboratory parameters in female HCV patients before DAA therapy. Table S2. Spearman correlation coefficients for the correlations of TGs (where species with identical numbers of carbon atoms or double bonds were grouped together) with age, BMI, the MELD score, viral load and routine laboratory parameters in male HCV patients before DAA therapy. Table S3. TGs (where species with identical numbers of carbon atoms or double bonds were grouped together) in the serum of males before DAA therapy stratified by fibrosis-4 score. Table S4. TGs (where species with identical numbers of carbon atoms were grouped together) in the serum of males without liver cirrhosis before DAA therapy stratified by genotype. Table S5. Spearman correlation coefficients for the correlations of TGs (where species with identical numbers of carbon atoms or double bonds were grouped together) with age, BMI, the MELD score, viral load and routine laboratory parameters in female HCV patients at the end of DAA therapy. Table S6. Spearman correlation coefficients for the correlations of TGs (where species with identical numbers of carbon atoms or double bonds were grouped together) with age, BMI, MELD score, viral load and routine laboratory parameters in male HCV patients at the end of DAA therapy. Table S7. Spearman correlation coefficients for the correlations of TGs (where species with identical numbers of carbon atoms or double bonds were grouped together) with the MELD score at the end of DAA therapy in males. Figure S1. Serum TG species in relation to body mass index (BMI) in female and male patients with chronic HCV. Figure S2. TGs stratified according to viral genotypes in male patients without liver cirrhosis at the end of the study. [file 12944_2022_1715_MOESM1_ESM.docx]

**Sex-specific changes of triglyceride profiles in liver cirrhosis and hepatitis C virus infection**

**Georg Peschel ^1,2^, Jonathan Grimm ^1^, Martina Müller ^1^, Marcus Höring ^3^, Sabrina Krautbauer ^3^, Kilian Weigand ^1, 4^, Gerhard Liebisch ^3^, and Christa Buechler^*,^ ^1^**

**Table S1.** Spearman correlation coefficients for the correlations of TGs (where species with identical numbers of carbon atoms or double bonds were grouped together) with age, BMI, MELD score, viral load and routine laboratory parameters in female HCV patients before DAA therapy. Significant correlations are in bold, * *P* < 0.05, ** *P* < 0.01, *** *P* < 0.001.

| **TG** | **C42** | **C44** | **C46** | **C48** | **C49** | **C50** | **C51** | **C52** | **C53** | **C54** | **C56** | **C58** | **DB0** | **DB1** | **DB2** | **DB3** | **DB4** | **DB5** | **DB6** | **DB7** | **DB8** | **Total** |
| --- | --- | --- | --- | --- | --- | --- | --- | --- | --- | --- | --- | --- | --- | --- | --- | --- | --- | --- | --- | --- | --- | --- |
| Age | 0.098 | 0.091 | 0.135 | 0.217 | 0.250 | 0.300 | 0.289 | 0.218 | 0.273 | 0.167 | 0.117 | 0.110 | 0.137 | 0.284 | 0.220 | 0.192 | 0.155 | 0.147 | 0.160 | 0.143 | 0.110 | 0.237 |
| BMI | 0.153 | 0.172 | 0.212 | 0.250 | 0.259 | 0.326 | 0.289 | 0.298 | 0.292 | 0.165 | 0.101 | 0.077 | 0.259 | 0.314 | 0.334 | 0.266 | 0.118 | 0.056 | 0.032 | 0.030 | 0.001 | 0.302 |
| MELD | -0.032 | -0.060 | -0.058 | -0.019 | -0.071 | 0.043 | -0.048 | -0.041 | -0.047 | -0.021 | -0.224 | -0.290 | -0.027 | 0.044 | -0.008 | -0.033 | -0.055 | -0.102 | -0.157 | -0.241 | -0.278 | -0.051 |
| Albumin | -0.169 | -0.156 | -0.146 | -0.176 | -0.115 | -0.210 | -0.113 | -0.097 | -0.097 | -0.097 | 0.047 | 0.113 | -0.172 | -0.207 | -0.153 | -0.094 | -0.045 | -0.010 | 0.035 | 0.106 | 0.143 | -0.142 |
| Bilirubin | -0.071 | -0.111 | -0.147 | -0.159 | -0.175 | -0.180 | -0.176 | -0.202 | -0.189 | -0.157 | -0.296 | -0.304 | -0.167 | -0.160 | -0.193 | -0.183 | -0.141 | -0.186 | -0.229 | -0.288 | -0.291 | -0.225 |
| INR | 0.010 | -0.002 | 0.012 | 0.039 | -0.014 | 0.069 | -0.030 | -0.070 | -0.056 | -0.084 | -0.243 | -0.284 | 0.018 | 0.071 | -0.007 | -0.061 | -0.096 | -0.129 | -0.175 | -0.236 | -0.279 | -0.051 |
| AST | 0.000 | 0.006 | 0.074 | 0.117 | 0.069 | 0.179 | 0.077 | 0.110 | 0.126 | 0.199 | -0.010 | 0.015 | 0.085 | 0.200 | 0.127 | 0.128 | 0.089 | 0.056 | 0.000 | -0.023 | -0.080 | 0.136 |
| ALT | -0.011 | 0.003 | 0.060 | 0.073 | 0.076 | 0.156 | 0.091 | 0.168 | 0.157 | 0.239 | 0.142 | 0.125 | 0.078 | 0.142 | 0.126 | 0.179 | 0.168 | 0.158 | 0.116 | 0.103 | 0.041 | 0.168 |
| Platelets | -0.033 | -0.024 | -0.063 | -0.110 | -0.128 | -0.140 | -0.111 | -0.001 | -0.083 | 0.031 | 0.094 | 0.101 | -0.038 | -0.127 | -0.015 | -0.005 | 0.009 | 0.040 | 0.059 | 0.083 | 0.120 | -0.031 |
| Creatinine | 0.160 | 0.145 | 0.136 | 0.155 | 0.172 | 0.211 | 0.228 | 0.208 | 0.211 | 0.174 | 0.114 | 0.021 | 0.181 | 0.217 | 0.239 | 0.183 | 0.100 | 0.079 | 0.049 | 0.008 | -0.014 | 0.188 |
| CRP | -0.230 | -0.236 | -0.253 | -0.206 | -0.188 | -0.088 | -0.115 | 0.075 | -0.009 | 0.042 | -0.031 | -0.080 | -0.252 | -0.160 | -0.016 | 0.059 | 0.057 | -0.004 | -0.038 | -0.042 | -0.001 | -0.028 |
| Leukocytes | 0.122 | 0.127 | 0.098 | 0.083 | 0.101 | 0.068 | 0.127 | 0.140 | 0.149 | 0.043 | 0.168 | 0.149 | 0.107 | 0.090 | 0.170 | 0.146 | 0.043 | 0.045 | 0.067 | 0.064 | 0.080 | 0.112 |
| Viral Load | 0.172 | 0.195 | 0.223 | 0.233 | 0.212 | 0.217 | 0.220 | 0.161 | 0.270 | 0.261 | 0.182 | 0.242 | 0.258 | 0.258 | 0.220 | 0.182 | 0.202 | 0.194 | 0.156 | 0.199 | 0.185 | 0.218 |
| HDL | -0.309 | -0.329 | **-0.353**  ***** | **-0.394**  ***** | **-0.344**  ***** | **-0.436**  ****** | **-0.374**  ***** | **-0.381**  ***** | **-0.366**  ***** | -0.301 | -0.155 | -0.085 | **-0.408**  ****** | **-0.438**  ****** | **-0.445**  ****** | **-0.372**  ***** | -0.214 | -0.143 | -0.088 | -0.064 | -0.004 | **-0.389**  ****** |
| LDL | 0.104 | 0.128 | 0.158 | 0.214 | 0.255 | 0.324 | 0.302 | **0.417**  ****** | 0.313 | 0.302 | **0.537**  ******* | **0.477**  ******* | 0.162 | 0.192 | **0.313**  ****** | **0.391**  ****** | **0.395**  ****** | **0.449**  ******* | **0.518**  ******* | **0.525**  ******* | **0.548**  ******* | **0.426**  ******* |

**Table S2.** Spearman correlation coefficients for the correlations of TGs (where species with identical numbers of carbon atoms or double bonds were grouped together) with age, BMI, MELD score, viral load and routine laboratory parameters in male HCV patients before DAA therapy. Significant correlations are in bold, * *P* < 0.05, ** *P* < 0.01, *** *P* < 0.001.

| **TG** | **C42** | **C44** | **C46** | **C48** | **C49** | **C50** | **C51** | **C52** | **C53** | **C54** | **C56** | **C58** | **DB0** | **DB1** | **DB2** | **DB3** | **DB4** | **DB5** | **DB6** | **DB7** | **DB8** | **Total** |
| --- | --- | --- | --- | --- | --- | --- | --- | --- | --- | --- | --- | --- | --- | --- | --- | --- | --- | --- | --- | --- | --- | --- |
| Age | 0.080 | 0.067 | 0.055 | 0.025 | 0.029 | 0.013 | -0.006 | -0.049 | -0.056 | -0.088 | -0.196 | -0.176 | 0.009 | 0.039 | 0.005 | -0.080 | -0.142 | -0.166 | -0.196 | -0.224 | -0.206 | -0.068 |
| BMI | 0.163 | 0.190 | 0.211 | 0.254 | 0.139 | 0.259 | 0.173 | 0.250 | 0.176 | 0.259 | 0.152 | 0.133 | 0.201 | 0.255 | 0.254 | 0.243 | 0.235 | 0.266 | 0.228 | 0.149 | 0.134 | 0.255 |
| MELD | 0.010 | 0.006 | 0.014 | -0.012 | -0.124 | -0.062 | -0.183 | -0.185 | -0.246 | -0.156 | **-0.326**  ****** | **-0.321**  ***** | -0.049 | -0.018 | -0.114 | -0.178 | -0.210 | -0.227 | -0.258 | **-0.307**  ***** | **-0.339**  ****** | -0.163 |
| Albumin | -0.210 | -0.186 | -0.153 | -0.119 | 0.020 | -0.040 | 0.091 | 0.143 | 0.186 | 0.137 | 0.263 | 0.263 | -0.153 | -0.121 | 0.033 | 0.159 | 0.201 | 0.206 | 0.216 | 0.274 | **0.290**  ***** | 0.096 |
| Bilirubin | 0.022 | -0.009 | -0.047 | -0.061 | -0.068 | -0.117 | -0.116 | -0.194 | -0.164 | -0.128 | -0.243 | -0.197 | -0.034 | -0.061 | -0.135 | -0.185 | -0.187 | -0.204 | -0.218 | -0.267 | -0.236 | -0.178 |
| INR | -0.059 | -0.060 | -0.052 | -0.061 | -0.184 | -0.086 | -0.228 | -0.200 | **-0.282**  ***** | -0.171 | **-0.342**  ****** | **-0.334**  ****** | -0.096 | -0.054 | -0.140 | -0.195 | -0.225 | -0.238 | -0.270 | **-0.313**  ***** | **-0.350**  ****** | -0.185 |
| AST | 0.012 | 0.005 | 0.011 | 0.047 | 0.037 | 0.059 | -0.016 | -0.078 | -0.070 | -0.063 | -0.140 | -0.080 | 0.035 | 0.073 | 0.008 | -0.073 | -0.132 | -0.144 | -0.144 | -0.160 | -0.160 | -0.044 |
| ALT | -0.159 | -0.132 | -0.100 | -0.015 | 0.038 | 0.042 | 0.019 | 0.001 | 0.015 | -0.021 | 0.067 | 0.126 | -0.073 | -0.006 | 0.017 | 0.009 | -0.012 | -0.004 | 0.029 | 0.083 | 0.104 | 0.007 |
| Platelets | -0.062 | -0.066 | -0.060 | -0.066 | 0.021 | -0.056 | 0.044 | 0.031 | 0.073 | -0.035 | 0.201 | 0.203 | -0.009 | -0.073 | -0.033 | 0.001 | 0.048 | 0.073 | 0.109 | 0.206 | 0.259 | -0.002 |
| Creatinine | 0.046 | 0.069 | 0.093 | 0.090 | 0.146 | 0.056 | 0.099 | 0.005 | 0.033 | -0.046 | -0.017 | -0.070 | 0.058 | 0.049 | -0.001 | 0.012 | 0.004 | -0.020 | -0.024 | -0.025 | -0.021 | 0.011 |
| CRP | -0.102 | -0.095 | -0.106 | -0.067 | 0.002 | -0.037 | -0.019 | -0.049 | -0.040 | -0.153 | -0.086 | -0.016 | -0.102 | -0.068 | -0.041 | -0.094 | -0.149 | -0.140 | -0.140 | -0.098 | -0.040 | -0.110 |
| Leukocytes | -0.071 | -0.026 | 0.006 | 0.078 | 0.129 | 0.123 | 0.167 | 0.138 | 0.173 | 0.062 | 0.169 | 0.178 | 0.085 | 0.118 | 0.163 | 0.092 | 0.036 | 0.040 | 0.048 | 0.097 | 0.127 | 0.116 |
| Viral Load | 0.056 | 0.103 | 0.110 | 0.123 | 0.166 | 0.100 | 0.169 | 0.096 | 0.154 | 0.155 | 0.154 | 0.142 | 0.109 | 0.112 | 0.091 | 0.115 | 0.146 | 0.163 | 0.178 | 0.164 | 0.127 | 0.137 |
| HDL | -0.163 | -0.230 | -0.282 | **-0.356**  ****** | -0.272 | **-0.389**  ****** | **-0.326**  ***** | **-0.373**  ****** | **-0.307**  ***** | **-0.456**  ******* | -0.251 | -0.137 | **-0.305**  ***** | **-0.392**  ****** | **-0.387**  ****** | **-0.408**  ******* | **-0.394**  ****** | **-0.397**  ****** | **-0.324**  ***** | -0.236 | -0.123 | **-0.413**  ******* |
| LDL | 0.102 | 0.155 | 0.232 | **0.289**  ***** | **0.411**  ******* | **0.325**  ***** | **0.415**  ******* | **0.384**  ****** | **0.417**  ******* | 0.204 | **0.453**  ******* | **0.423**  ******* | 0.158 | 0.258 | **0.327**  ***** | **0.342**  ****** | **0.306**  ***** | **0.323**  ***** | **0.345**  ****** | **0.406**  ******* | **0.421**  ******* | **0.350**  ****** |

**Table S3.** TGs (where species with identical numbers of carbon atoms or double bonds were grouped together) in the serum of males before DAA therapy stratified by fibrosis-4 score. * *P* < 0.05.

| **TG nmol/ml** |  | **C56** | **DB7** | **DB8** |
| --- | --- | --- | --- | --- |
| **No Fibrosis** | Median | 65.2* | 29.8* | 11.4* |
|  | Minimum | 22.7 | 6.4 | 2.1 |
|  | Maximum | 149.5 | 74.6 | 47.2 |
| **Inconclusive Fibrosis** | Median | 63.7 | 24.5 | 8.6 |
|  | Minimum | 8.8 | 1.8 | 0.8 |
|  | Maximum | 244.1 | 118.3 | 60.5 |
| **Fibrosis** | Median | 41.5* | 16.7* | 6.3* |
|  | Minimum | 4.9 | 1.5 | 0.1 |
|  | Maximum | 111.8 | 68.7 | 35.4 |

**Table S4.** TGs (where species with identical numbers of carbon atoms were grouped together) in the serum of males without liver cirrhosis before DAA therapy stratified by genotype. * *P* < 0.05, ** *P* < 0.01.

| **TG nmol/ml** |  | **C49** | **C51** | **C53** | **C56** |
| --- | --- | --- | --- | --- | --- |
| **Genotype 1a** | Median | 17.6** | 32.2* | 20.3* | 65.7 |
|  | Minimum | 5.5 | 14.4 | 11.8 | 39.4 |
|  | Maximum | 50.2 | 81.8 | 47.9 | 149.5 |
| **Genotype 1b** | Median | 14.6 | 30.8 | 23.3 | 78.6* |
|  | Minimum | 2.7 | 7.6 | 6.5 | 25.5 |
|  | Maximum | 141.3 | 217.8 | 119.6 | 244.1 |
| **Genotype 3a** | Median | 7.1** | 17.7* | 13.1* | 47.0* |
|  | Minimum | 0.2 | 3.8 | 3.6 | 8.8 |
|  | Maximum | 36.8 | 58.4 | 29.8 | 97.5 |
| **Rare Genotypes** | Median | 16.6 | 31.6 | 19.7 | 75.0 |
|  | Minimum | 13.0 | 24.7 | 14.1 | 51.7 |
|  | Maximum | 41.2 | 69.3 | 38.4 | 111.8 |

**Table S5.** Spearman correlation coefficients for the correlations of TGs (where species with identical numbers of carbon atoms or double bonds were grouped together) with age, BMI, MELD score, viral load and routine laboratory parameters in female HCV patients at the end of DAA therapy. Significant correlations are in bold, * *P* < 0.05, ** *P* < 0.01.

| **TG** | **C42** | **C44** | **C46** | **C48** | **C49** | **C50** | **C51** | **C52** | **C53** | **C54** | **C56** | **C58** | **DB0** | **DB1** | **DB2** | **DB3** | **DB4** | **DB5** | **DB6** | **DB7** | **DB8** | **Total** |
| --- | --- | --- | --- | --- | --- | --- | --- | --- | --- | --- | --- | --- | --- | --- | --- | --- | --- | --- | --- | --- | --- | --- |
| Age | **0.352**  ***** | **0.365**  ***** | **0.388**  ****** | **0.395**  ****** | **0.387**  ****** | **0.383**  ****** | **0.365**  ***** | 0.201 | 0.252 | 0.093 | 0.124 | 0.104 | **0.415**  ****** | **0.431**  ****** | 0.283 | 0.164 | 0.133 | 0.190 | 0.193 | 0.177 | 0.100 | 0.273 |
| BMI | 0.329 | 0.294 | 0.255 | 0.218 | 0.165 | 0.202 | 0.178 | 0.160 | 0.150 | -0.010 | 0.132 | 0.149 | 0.312 | 0.242 | 0.167 | 0.119 | 0.040 | 0.049 | 0.067 | 0.080 | 0.112 | 0.171 |
| MELD | 0.175 | 0.139 | 0.127 | 0.111 | 0.085 | 0.141 | 0.077 | 0.054 | 0.036 | 0.073 | -0.041 | -0.079 | 0.180 | 0.200 | 0.096 | 0.039 | 0.023 | 0.003 | -0.027 | -0.051 | -0.092 | 0.092 |
| Albumin | -0.087 | -0.023 | 0.017 | 0.048 | 0.116 | 0.043 | 0.124 | 0.074 | 0.170 | 0.092 | 0.150 | 0.162 | -0.078 | -0.052 | 0.088 | 0.087 | 0.081 | 0.099 | 0.108 | 0.128 | 0.095 | 0.059 |
| Bilirubin | 0.176 | 0.119 | 0.106 | 0.091 | 0.015 | 0.063 | -0.048 | -0.087 | -0.126 | -0.108 | -0.176 | -0.220 | 0.147 | 0.146 | 0.029 | -0.089 | -0.142 | -0.158 | -0.174 | -0.200 | -0.202 | -0.041 |
| INR | 0.139 | 0.126 | 0.114 | 0.085 | 0.080 | 0.084 | 0.032 | -0.018 | -0.034 | 0.013 | -0.124 | -0.174 | 0.175 | 0.157 | 0.015 | -0.028 | -0.029 | -0.049 | -0.078 | -0.122 | -0.179 | 0.027 |
| AST | 0.272 | 0.227 | 0.217 | 0.192 | 0.131 | 0.230 | 0.120 | 0.094 | 0.111 | 0.072 | 0.007 | -0.038 | 0.282 | 0.271 | 0.205 | 0.080 | 0.001 | 0.000 | -0.008 | -0.027 | -0.061 | 0.152 |
| ALT | 0.211 | 0.196 | 0.177 | 0.127 | 0.121 | 0.144 | 0.091 | 0.024 | 0.056 | -0.001 | 0.026 | 0.002 | 0.227 | 0.182 | 0.097 | 0.004 | -0.052 | -0.024 | 0.008 | 0.009 | 0.001 | 0.069 |
| Platelets | -0.186 | -0.172 | -0.174 | -0.154 | -0.175 | -0.119 | -0.103 | 0.057 | -0.024 | 0.103 | 0.160 | 0.173 | -0.187 | -0.177 | -0.033 | 0.049 | 0.099 | 0.131 | 0.153 | 0.181 | 0.217 | -0.012 |
| Creatinine | 0.075 | 0.035 | 0.022 | 0.031 | 0.025 | 0.044 | 0.069 | 0.041 | 0.083 | 0.048 | 0.049 | 0.056 | 0.055 | 0.035 | 0.035 | 0.036 | 0.083 | 0.090 | 0.090 | 0.122 | 0.126 | 0.043 |
| CRP | -0.008 | -0.020 | -0.052 | -0.042 | -0.007 | -0.007 | 0.009 | -0.032 | -0.004 | -0.101 | -0.115 | -0.173 | 0.055 | 0.029 | 0.003 | -0.081 | -0.106 | -0.137 | -0.145 | -0.150 | -0.155 | -0.042 |
| Leukocytes | -0.104 | -0.063 | -0.030 | 0.037 | 0.029 | 0.080 | 0.082 | 0.112 | 0.093 | 0.017 | 0.168 | .245^*^ | -0.079 | 0.014 | 0.122 | 0.088 | 0.038 | 0.056 | 0.083 | 0.147 | 0.217 | 0.073 |
| HDL | -0.216 | -0.178 | -0.204 | -0.272 | -0.247 | **-0.359**  ***** | -0.320 | **-0.395**  ****** | -0.319 | **-0.356**  ***** | -0.333 | -0.285 | -0.277 | -0.328 | **-0.385**  ***** | **-0.374**  ***** | **-0.349**  ***** | **-0.365**  ***** | **-0.352**  ***** | **-0.336**  ***** | -0.296 | **-0.402**  ****** |
| LDL | 0.152 | 0.196 | 0.238 | 0.269 | 0.278 | 0.278 | 0.300 | 0.288 | 0.281 | 0.200 | 0.317 | 0.282 | 0.166 | 0.197 | 0.306 | 0.272 | 0.255 | 0.279 | 0.286 | 0.293 | 0.259 | 0.286 |

**Table S6.** Spearman correlation coefficients for the correlations of TGs (where species with identical numbers of carbon atoms or double bonds were grouped together) with age, BMI, MELD score, viral load and routine laboratory parameters in male HCV patients at the end of DAA therapy. Significant correlations are in bold, * *P* < 0.05, ** *P* < 0.01, *** *P* < 0.001.

| **TG** | **C42** | **C44** | **C46** | **C48** | **C49** | **C50** | **C51** | **C52** | **C53** | **C54** | **C56** | **C58** | **DB0** | **DB1** | **DB2** | **DB3** | **DB4** | **DB5** | **DB6** | **DB7** | **DB8** | **Total** |
| --- | --- | --- | --- | --- | --- | --- | --- | --- | --- | --- | --- | --- | --- | --- | --- | --- | --- | --- | --- | --- | --- | --- |
| Age | -0.035 | -0.023 | -0.001 | -0.022 | 0.022 | -0.021 | -0.012 | -0.095 | -0.051 | -0.152 | -0.254 | -0.236 | -0.037 | -0.009 | -0.022 | -0.109 | -0.170 | -0.206 | -0.228 | -0.231 | -0.246 | -0.106 |
| BMI | 0.098 | 0.092 | 0.137 | 0.152 | 0.083 | 0.204 | 0.128 | 0.184 | 0.122 | 0.177 | 0.097 | 0.038 | 0.118 | 0.181 | 0.178 | 0.183 | 0.176 | 0.182 | 0.144 | 0.067 | 0.032 | 0.171 |
| MELD | -0.217 | -0.252 | -0.258 | -0.263 | -0.229 | -0.202 | -0.245 | -0.261 | -0.262 | -0.229 | **-0.309**  ***** | -0.272 | -0.203 | -0.210 | -0.211 | -0.260 | **-0.287**  ***** | **-0.295**  ***** | **-0.308**  ***** | **-0.320**  ****** | **-0.332**  ****** | -0.267 |
| Albumin | 0.024 | 0.073 | 0.131 | 0.173 | 0.148 | 0.167 | 0.170 | 0.242 | 0.214 | 0.208 | **0.331**  ****** | **0.305**  ***** | 0.060 | 0.093 | 0.185 | 0.247 | 0.266 | **0.280**  ***** | **0.321**  ***** | **0.348**  ****** | **0.336**  ****** | 0.235 |
| Bilirubin | -0.068 | -0.111 | -0.116 | -0.141 | -0.135 | -0.161 | -0.164 | -0.248 | -0.227 | -0.236 | -0.270 | -0.279 | -0.056 | -0.133 | -0.205 | -0.246 | -0.245 | -0.248 | -0.239 | -0.257 | -0.252 | -0.236 |
| INR | -0.136 | -0.169 | -0.182 | -0.224 | -0.213 | -0.191 | -0.252^*^ | -0.288  * | -0.271 | -0.252 | **-0.353**  ****** | -0.275 | -0.141 | -0.161 | -0.195 | **-0.284**  ***** | **-0.336**  ****** | **-0.347**  ****** | **-0.366**  ****** | **-0.348**  ****** | **-0.362**  ****** | **-0.282**  ***** |
| AST | -0.152 | -0.183 | -0.183 | -0.196 | -0.192 | -0.132 | -0.183 | -0.160 | -0.172 | -0.095 | -0.158 | -0.105 | -0.178 | -0.166 | -0.129 | -0.150 | -0.168 | -0.183 | -0.185 | -0.162 | -0.168 | -0.159 |
| ALT | 0.046 | 0.038 | 0.052 | 0.084 | 0.043 | 0.149 | 0.093 | 0.185 | 0.123 | 0.213 | **0.284**  ***** | **0.341**  ****** | 0.033 | 0.082 | 0.152 | 0.195 | 0.193 | 0.199 | 0.230 | **0.290**  ***** | **0.284**  ***** | 0.186 |
| Platelets | 0.068 | 0.097 | 0.094 | 0.113 | 0.092 | 0.072 | 0.123 | 0.139 | 0.134 | 0.111 | 0.240 | 0.208 | 0.065 | 0.044 | 0.076 | 0.135 | 0.174 | 0.203 | 0.246 | 0.262 | 0.279  * | 0.144 |
| Creatinine | -0.128 | -0.141 | -0.149 | -0.127 | -0.052 | -0.076 | -0.035 | -0.002 | -0.017 | 0.012 | -0.006 | -0.038 | -0.151 | -0.130 | -0.069 | -0.012 | 0.020 | 0.011 | -0.002 | -0.043 | -0.036 | -0.035 |
| CRP | -0.001 | -0.022 | -0.040 | -0.088 | -0.091 | -0.091 | -0.114 | -0.100 | -0.104 | -0.050 | -0.171 | -0.177 | 0.001 | -0.046 | -0.099 | -0.099 | -0.082 | -0.099 | -0.146 | -0.191 | -0.186 | -0.104 |
| Leukocytes | 0.031 | 0.055 | 0.057 | 0.082 | 0.068 | 0.083 | 0.100 | 0.159 | 0.112 | 0.141 | 0.165 | 0.128 | 0.060 | 0.091 | 0.114 | 0.149 | 0.146 | 0.160 | 0.178 | 0.159 | 0.154 | 0.156 |
| HDL | -0.153 | -0.171 | -0.201 | -0.267 | -0.238 | **-0.357**  ****** | **-0.295**  ***** | **-0.363**  ****** | **-0.310**  ***** | **-0.301**  ***** | -0.266 | -0.213 | -0.235 | **-0.300**  ***** | **-0.363**  ****** | **-0.347**  ****** | **-0.310**  ***** | **-0.285**  ***** | -0.259 | -0.213 | -0.194 | -**0.350**  ****** |
| LDL | 0.244 | **0.333**  ****** | **0.391**  ****** | **0.480**  ******* | **0.484**  ******* | **0.527**  ******* | **0.558**  ******* | **0.581**  ******* | **0.588**  ******* | **0.506**  ******* | **0.546**  ******* | **0.489**  ******* | **0.313**  ***** | **0.450**  ******* | **0.561**  ******* | **0.586**  ******* | **0.547**  ******* | **0.524**  ******* | **0.534**  ******* | **0.525**  ******* | **0.486**  ******* | **0.586**  ******* |

**Table S7.** Spearman correlation coefficients for the correlations of TGs (where species with identical numbers of carbon atoms or double bonds were grouped together) with the MELD score at the end of direct-acting antiviral (DAA) therapy in males. Associations were controlled for LDL levels.

| **TG** | **C56** | **DB4** | **DB5** | **DB6** | **DB7** | **DB8** |
| --- | --- | --- | --- | --- | --- | --- |
| MELD | -0.080 | -0.061 | -0.081 | -0.101 | -0.110 | -0.113 |

**
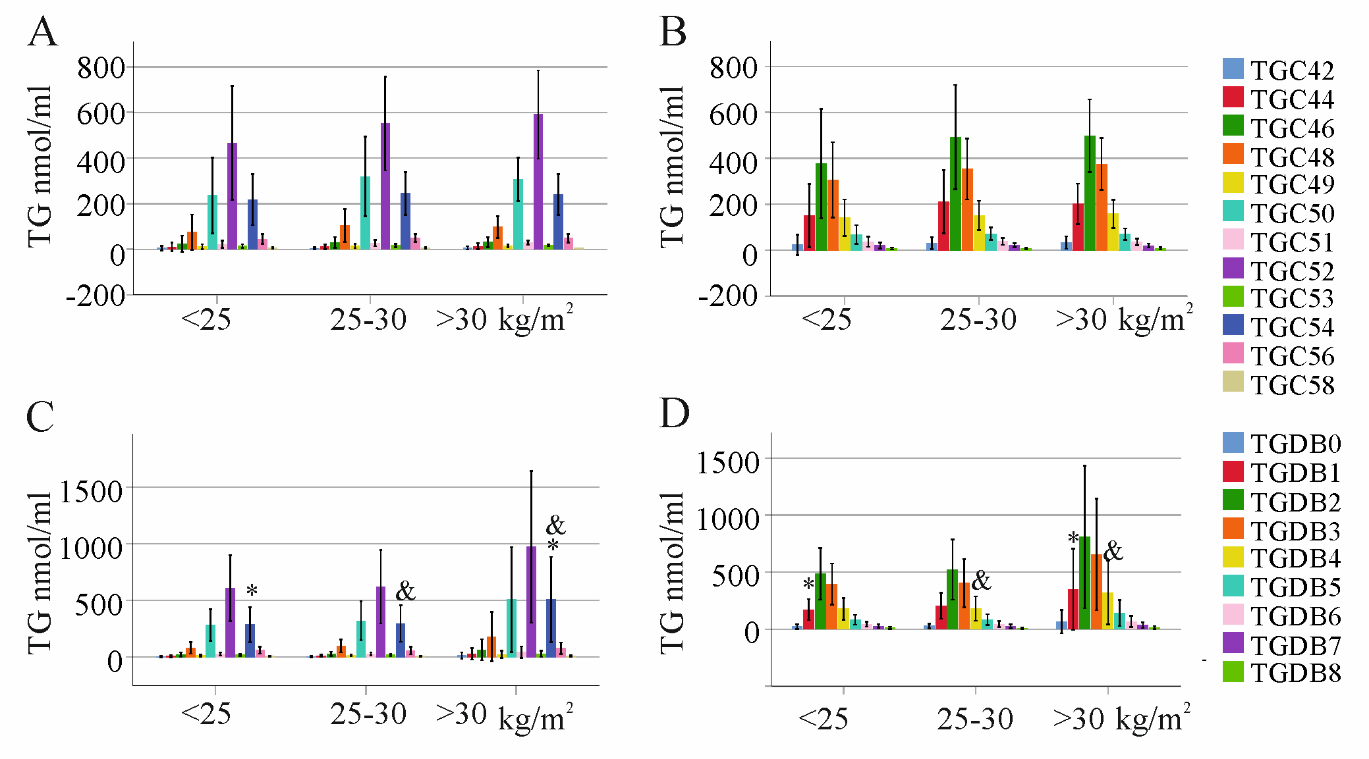
**

**Figure S1.** Serum TG species in relation to body mass index (BMI) in female and male patients with chronic HCV. (**a**) TG species with identical numbers of carbon atoms were grouped together in female patients stratified for BMI; (**b**) TG species with identical numbers of double bonds were grouped together in female patients stratified for BMI; (**c**) TG species with identical numbers of carbon atoms were grouped together in male patients stratified for BMI; (**d**) TG species with identical numbers of double bonds were grouped together in male patients stratified for BMI. * p < 0.05, ^&^ p < 0.05.

**
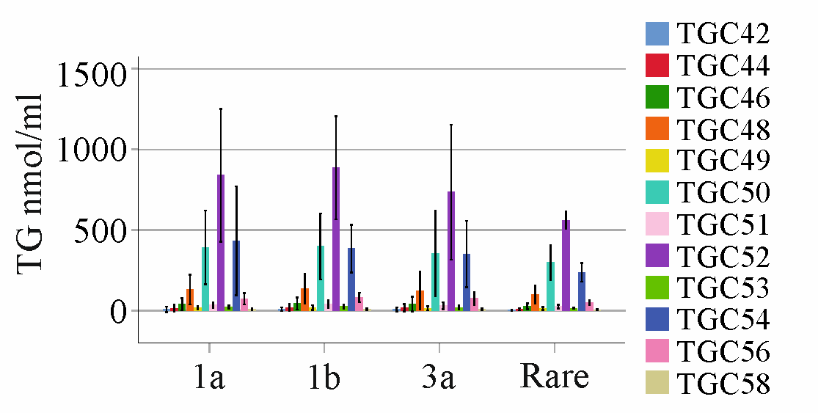
**

**Figure S2.** TGs stratified according to viral genotypes in male patients without liver cirrhosis at the end of the study**.** (**a**) TGs with identical numbers of carbon atoms in serum of males with different viral genotypes.
